# Determining Modern Marketing Mix Elements and Their Measurement Indicators for Selling Cosmetic and Health Products in Iranian Pharmacies: A Descriptive-Analytical Cross-sectional Study

**DOI:** 10.5812/ijpr-166280

**Published:** 2026-02-12

**Authors:** Reza Yaghoubi Nezhad, Majid Annabi, Amin Akbari, Yasaman Bastami, Mahdi Maleki Zadeh

**Affiliations:** 1 Student Research Committee, Faculty of Pharmacy , Tehran University of Medical Science , Islamic Azad university , Tehran , Iran; 2 Department of Pharmacoeconomics, Faulty of Pharmacy, Tehran Medical Sciences, Islamic Azad university, Tehran, Iran; 3 Health Management and Economics Research Center, Health Management Research Institute, Iran University of Medical Sciences, Tehran, Iran; 4 Student Research Committee, Faculty of Pharmacy , Tehran University of Medical Science , Islamic Azad University , Tehran , Iran

**Keywords:** Modern Marketing Mix, Marketing Elements, Cosmetic-Health Products, Pharmacy

## Abstract

**Background:**

The cosmetic and health products market is a competitive market. Traditional marketing elements do not respond to the market, and the use of modern marketing mixes is necessary for sales success. With the increase in the number of pharmacies in Iran, they are forced to use modern marketing mix elements for this purpose

**Objectives:**

The present study aims to identify and define modern marketing mix elements and their measurement indicators that are effective on the sale of cosmetic and health products.

**Methods:**

The research method is conducted as a descriptive-analytical cross-sectional study. The statistical population consists of two groups. The first group is experts to determine modern marketing mix elements and the validity of their items, who have been identified and selected with the Snowball method. The second group is customers in Iranian pharmacies. In the experts' point of view, mix elements are determined using the fuzzy Delphi method, and the validity of their elements and items is determined using content and face validity assessment. Subsequently, the effect of the determined factors in Iranian pharmacies is determined using factor analysis of structural equations, and the validity and reliability of their measurement indicators are confirmed (Cronbach's alpha and composite reliability obtained were higher than 0.7).

**Results:**

The results indicated that out of the twelve elements of the modern marketing mixes, the seven elements of price, quality, attractiveness, brand image, emotions, advertising, and customer trust are effective elements on the sale of cosmetic and health products, for which measurement indicators have been proposed in the market. The model showed an acceptable fit with CFI values above 0.93, indicating good consistency between the model and the observed data, and path analysis showed that price (β = 0.78), product quality (β = 0.75), and emotional appeal (β = 0.75) had the strongest positive effects on cosmetic and hygiene product sales.

**Conclusions:**

This study highlights that traditional marketing mix elements are insufficient to explain customer behavior in the competitive cosmetic and hygienic products market in Iran. Instead, seven modern marketing mix elements (price, quality, attractiveness, brand image, emotions, advertising, and customer trust) play a crucial role in shaping customer satisfaction and purchasing decisions.

## 1. Background

The marketing mix refers to controllable elements such as product, price, place, and promotion that companies combine to influence consumer demand (1). Although the 4Ps remain the foundation, scholars have expanded the model to include consumer-oriented and industry-specific dimensions, leading to variations such as 7Ps, 4C, and 12Ps (2). Cosmetic and health products have economic, social, and cultural significance for consumers and represent a competitive market with diverse domestic and international brands (3, 4). In Iran, with a sharp rise in the number of pharmacies, they now rely increasingly on cosmetic and health product sales in addition to medicines (5). This shift highlights the need to adopt modern marketing mix strategies to sustain growth and meet customer expectations (6). Recent analyses of cosmetic products in the Iranian market have highlighted inconsistencies between labeled and actual compositions of ingredients (e.g., quantitative analysis of skin-lightening creams by HPLC). Such findings reflect broader challenges in the cosmetic and healthcare products industry, emphasizing the importance of product reliability and transparency (key components influencing consumer trust and purchase decisions in this market) (7).

## 2. Objective

In this study, the modern marketing mix is conceptualized through a 12P framework, including price, product quality, place, advertising, people, politics, process, physical attractiveness, collaboration, emotions and perceptions, program, and source/brand. This framework provides a comprehensive basis for examining contemporary marketing practices and was used as the initial reference model for expert evaluation. Accordingly, this study addresses the key question: Which modern marketing mix elements are most important in selling cosmetic and health products in Iranian pharmacies?

## 3. Methods

### 3.1. Research Literature

 The research literature consists of four sections, which are described below. These four sections are:

(1) Marketing mixes

(2) Modern marketing mixes

(3) Application and advantage of marketing mix in selling cosmetic and health products

(4) Research history and research gaps

#### 3.1.1. Marketing Mix

 In 1960, the American Marketing Association defined the first official definition of marketing as “the conduct of business activities that direct the flow of goods and services from producer to consumer” (8). The concept of marketing mix was first introduced by Borden in 1964, but the elements introduced in this concept, including product, price, advertising, and place, were introduced by McCarthy (2). In other words, the marketing decision-making variable, conceptualized through marketing mix models, provides a framework upon which companies formulate and align their marketing activities (9, 10).

#### 3.1.2. Modern Marketing Mixes

The marketing mix is not a scientific theory, but a conceptual framework that determines the key decisions of managers in configuring their offerings to meet consumer needs (10). The role of the price element has long been very important in the selection and purchase of products by customers; and then, other elements have been proposed beside this element and expanded from a single P to the 4Ps (11). Whereas the 4Ps still have significant virality, most marketing thinkers agree that these elements should be developed to include all dimensions of the organization, the market, and the people as customers (12) because there are environments in which the combinations of elements should be adapted to the specific requirements of that environment. Bitner and Boom in 1981 presented a model in which, in addition to price, products, place, and advertising, items including cooperation, process, and physical evidence are also mentioned to support this claim (13). Salman et al. examined all marketing elements in previous research and presented a conceptual model of the modern marketing mix which includes Price, Quality, Place, Advertising, Trust, Politics, Process, Physical Attractiveness, Collaboration, Emotions, Program, Source, and Brand (14). In this study, the term "modern marketing" refers not to digital marketing, but to the conceptual expansion of the classical 4Ps framework into contemporary multidimensional models (7Ps-12Ps), which integrate additional elements such as people, process, physical evidence, and performance (15-17).

#### 3.1.3. Applications and Advantages of Modern Marketing Mix in Selling Cosmetics

The cosmetics industry requires careful market analysis, segmentation, and targeting to align with consumer preferences (5). The marketing mix serves both as a tool for product positioning and as a framework for evaluating sales strategies (18). Its advantages include adapting inventory allocation to diverse customer demands (19). By extending beyond the traditional 4Ps to modern models such as 12Ps, cosmetic companies can design strategies to enhance sales and competitiveness (20).

#### 3.1.4. Research Background and Research Gaps

Previous studies have highlighted the influence of various factors such as brand image, quality, price, emotions, advertising, and trust on cosmetic product sales across different contexts (20-22). However, most focused on specific elements or limited populations, without integrating a comprehensive framework. To date, no study has systematically identified and validated modern marketing mix elements and their measurement indicators in the context of Iranian pharmacies. This research addresses that gap (23).

### 3.2. Research Method

**Figure 1.**
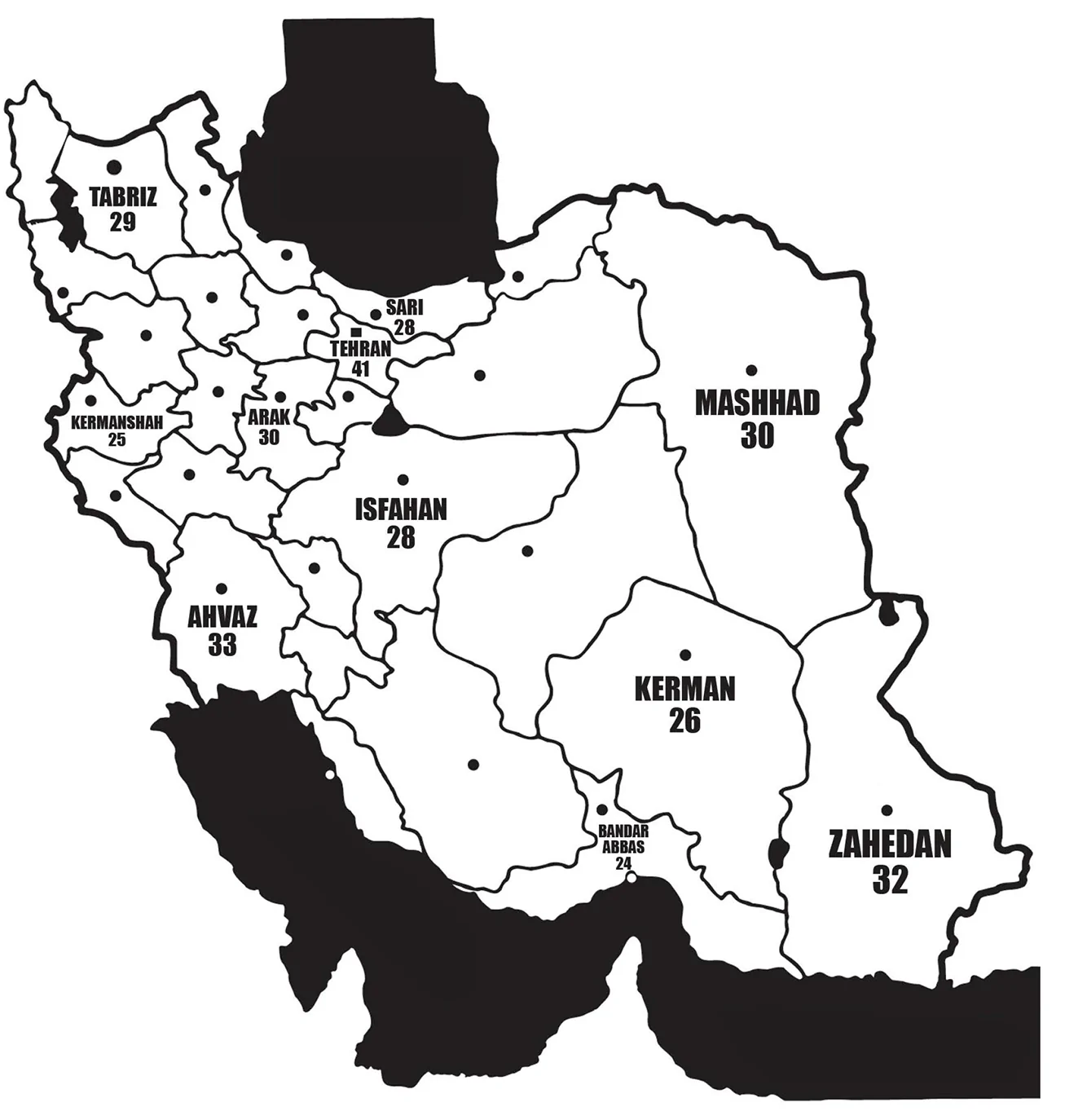
Geographical distribution of the collected questionnaires

The research method is applied in terms of purpose; and it is descriptive in terms of method. The statistical population includes two groups in the qualitative and quantitative sections. The first group, experts, includes CEOs and chairs of the board of directors of companies supplying cosmetic and health products and professors in the field of pharmacy who have the necessary knowledge and experience. The conditions of expertise are related to two parts based on the research subject. First is the knowledge and experience of the expert in the knowledge of cosmetic and health products and second, the knowledge and experience of selling cosmetic and health products in Iran. For each part, the condition of years of experience according to the Likert scale has been considered (this classification is shown in Appendix 5 in Supplementary File) in the attached file, where the condition for selecting an expert is to have a minimum score of 7 out of 10). A semi-expert questionnaire tool is used to collect data from experts. Given the collected data, experts who have the required score of the expected conditions are identified using the Snowball method. In a way that the interview started with two experts whom the researcher knew and the other one is introduced by experts 1 and 2 and this process continued until the required reliability is obtained using the P-Scott method. In the P-Scott method, the percentage of agreement in the interview must be at least 80 percent. From the interview of the seventh person, the percentage of agreement is 82 percent. On the other hand, the interviews continued until the tenth person, when no new factors and categories are gained and the Scott coefficient reached 94 percent. This group is used in the fuzzy Delphi method with a semi-specialized questionnaire tool to determine the modern marketing mix elements that are effective in sales of cosmetic and health products and then to determine the validity of the questionnaire questions obtained from the effective elements. The second group of the statistical population is all customers of cosmetic products in Iranian pharmacies, whose opinions are used to determine the effect of the questions in measuring the effect of selected marketing mix elements on the sale of cosmetic products. Based on the Morgan table, the need for 384 samples is determined as respondents to the questionnaires. Sampling is carried out by the stratified-convenience method. Considering the possibility of non-return of the questionnaires, 550 questionnaires are distributed in different regions of Iran. In each region, 50 questionnaires are distributed in pharmacies. After the follow-ups, 390 questionnaires are returned and 62 questionnaires are distorted and have errors, and finally 328 healthy questionnaires are used for data analysis. Data collection was performed between March 2025 and July 2025. Questionnaires were administered to customers over 18 years of age who purchased cosmetic and health products at community pharmacies across 11 geographically representative regions of Iran (provincial centers included: Sari, Tabriz, Mashhad, Bandar Abbas, Ahvaz, Zahedan, Kermanshah, Kerman, Tehran, Arak, Isfahan). To minimize potential selection bias, data were collected at different times and days in each region, and demographic characteristics of respondents were reviewed to confirm diversity across regions. The geographical distribution of the collected questionnaires is shown in Figure 1. 

The distributed questionnaire consists of 35 questions with a 5-point Likert scale that includes questions for seven elements of the modern marketing mix that affect the sales of cosmetic products in Iranian pharmacies. The first part of the questionnaire is dedicated to obtaining basic and demographic information of the respondents (References related to the design of questions for each factor in the questionnaire are given in Appendix 4 in Supplementary File.)

### 3.3. Validity and Reliability of the Measurement Tool 

The Content Verification method is used to determine the validity of the questionnaire. This content review and approval is confirmed by experts. The Content Validity Ratio Index (CVR) or Lawshe's content validity is used to measure the validity of the questionnaire. The purpose of the research is first explained to the experts to examine this; then, they are asked to rate each of the items.

## 4. Results

To identify the modern marketing mix elements effective in cosmetic and health product sales, opinions from two groups of experts and customers were collected and analyzed. First, the elements are selected by experts and then the effect of these elements in the real environment is fact-checked from the customers' point of view.

### 4.1. Determining Modern Marketing Mix Elements Effective on Cosmetic-Health Products Sales from the Experts' Point of View

Two steps are used to identify the effective elements in the modern marketing mix. The first step is to prepare the modern marketing mix elements table from the conceptual model of Salman et al. 2017 and the second step is to use the experts' opinion with the fuzzy Delphi method to determine the appropriate and necessary elements effective on cosmetic-health products sales (14). After presenting the elements to the experts, their opinions are collected using the fuzzy Delphi method. In the first stage, 5 elements are found to be ineffective for selling cosmetic and health products, and the survey went to the second step; and in the second step, the remaining seven elements are approved with a consensus of over 70 percent (the complete statistical table of the frequency of Likert scale responses to the questionnaire in the Fuzzy Delphi Method is presented in the attached file, Appendix 1, along with explanations.)

### 4.2. Study of Modern Marketing Mix Elements Effect on the Sales of Cosmetic-Health Products from the Customers' Point of View 

Confirmatory factor analysis was conducted using data collected from a customer survey to validate the identified elements. Data were collected from customers in eleven regions of Iran through a questionnaire, and confirmatory factor analysis in structural equations and LISREL software was used to confirm the modern marketing mix elements, and Cronbach's alpha coefficient was used to confirm the questions with SPSS software. In order to apply confirmatory factor analysis, it is necessary to confirm the fit of the model indices, which are examined below and the results are shown in Table 1. 

**Table 1.** Result of Fit Indices of the Model ^a^

R	Modern Marketing Mix Elements	χ^2^/df	RMSEA	CFI	NFI	GFI
**1**	Brand image	2.658	0.068	0.945	0.954	0.921
**2**	Attractiveness	1.252	0.025	0.977	0.989	0.923
**3**	Emotions	1.46	0.035	0.957	0.944	0.917
**4**	Quality/product	2.327	0.061	0.934	0.984	0.922
**5**	Trust	1.656	0.043	0.955	0.969	0.915
**6**	Advertising	2.787	0.073	0.933	0.923	0.913
**7**	Price	2.107	0.056	0.968	0.943	0.918

^a^ Source: Research findings.

**Figure 2.**
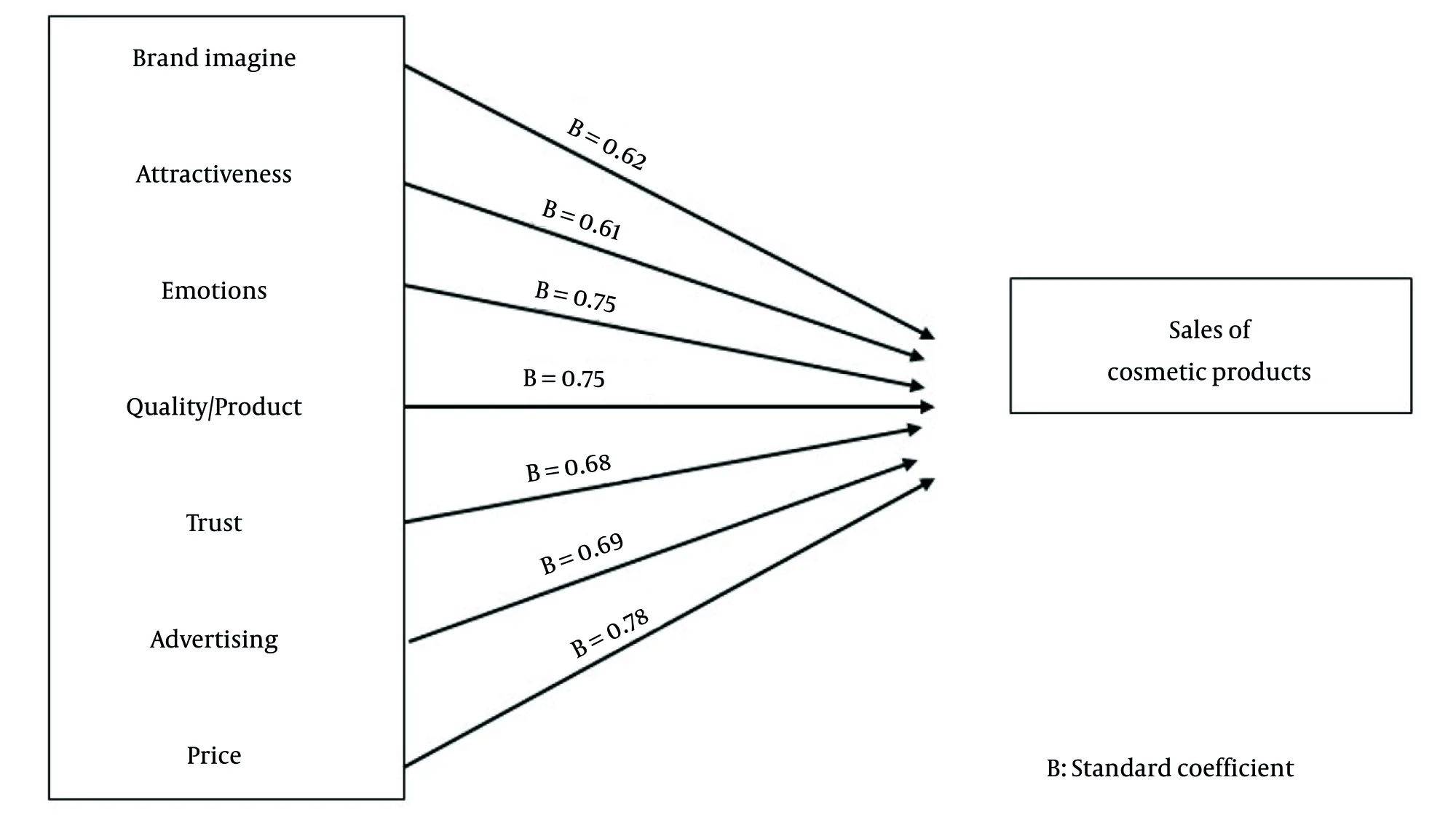
Results of structural equations analysis for modern marketing mix elements effective on the sales of cosmetic products

According to the fit indices, it is observed that there is a good fit and the results of structural equations can be used. For confirmatory factor analysis, the size of the model parameters is obtained using the LISREL software. In order to examine the significance of the proposed factor loading, *t*-score is used and the results are presented in Figure 2. 

Potential confounding variables, including participants’ age, gender, education level, and purchasing experience, were statistically tested. The analyses showed no significant associations between these variables and the main study constructs, indicating minimal confounding effects in the results (the table and explanations related to the statistical test of confounding variables are given in the attached file, Appendix 3 in Supplementary File).

### 4.3. Determining Measurement Indicators of New Mix Elements Effective on the Sales of Cosmetic and Health Products 

Subsequent to confirming the mix elements of marketing that are effective on the sales of cosmetic and health products, it is essential to determine their measurement indicators, which is done by examining their validity and reliability.

### 4.4. Determining the Validity of Measurement Indicators from the Experts' Point of View 

For each element of the modern marketing mix that affects the sales of cosmetic products, a measurement plan has been designed, which requires validation of customer opinions to gauge their validity. For this purpose, CVR is determined from 10 experts' point of view, a panel of domain experts was consulted, including academic faculty members in pharmaceutical and marketing sciences, professionals with expertise in the cosmetics industry, and pharmacists employed in cosmetic product companies. According to the collection of opinions of 10 experts, the questions whose CVR index was lower than 0.62 were eliminated (questions 5 and 11), and the remaining questions were approved.

### 4.5. Measuring the Reliability of the Questionnaire

#### 4.5.1. Reliability According to Cosmetic and Health Experts

The test-retest reliability method is applied to measure the reliability according to experts. In this study, four interviews are randomly selected and each of them is coded twice within a 30-day time interval. After that, the codes identified in the two times are compared for each interview and a consistency index is calculated for each interview based on the amount of agreement and disagreement. In each interview, codes that are similar in the two times are labeled as agreement and dissimilar codes are labeled as disagreement. The test-retest reliability of the interviews conducted in the present study is 65 percent, and since this value is above 60 percent, the reliability of the items is confirmed (the test-retest reliability from the experts' perspective is fully presented in the attached file, Appendix 2 in Supplementary File).

#### 4.5.2. Reliability in Terms of Cosmetic and Health Products Customers

Both Cronbach's alpha and composite reliability methods have been used to better measure reliability. If the Cronbach's alpha and composite reliability values for each construct (variable) are above 0.7, it indicates appropriate internal consistency for the measurement model. The values of them are shown in Table 2. 

**Table 2.** Cronbach's Alpha Coefficients and Composite Reliability

R	Modern Marketing Mix Elements	Cronbach's Alpha	Composite Reliability
**1**	Brand image	0.718	0.828
**2**	Attractiveness	0.802	0.844
**3**	Emotions	0.762	0.792
**4**	Quality/product	0.903	0.922
**5**	Trust	0.845	0.877
**6**	Advertising	0.724	0.769
**7 **	Price	0.866	0.881

#### 4.5.3. Limitations

Despite efforts to increase national representation through regional stratification and long data collection periods, this study has certain limitations. The sample size, although statistically sufficient for an unincorporated population, we encourage future studies to use larger probability-based samples covering all provinces to improve generalizability and deepen regional level insights.

## 5. Discussion and Conclusions

Cosmetic and health products have established a major portion of pharmacy sales in recent years. Various marketing elements play different roles in selling these products to successfully sell cosmetic and health products in this market, and the traditional marketing elements are not corresponding to the needs and diversity of customers' demands. The use of modern marketing mixes is essential for the success of selling these products in Iranian pharmacies given that the number of pharmacies in Iran has increased considerably in recent years. This study has identified modern marketing mix elements to help in choosing a marketing strategy in Iranian pharmacies. Of the twelve elements of the modern marketing mix, experts have suggested seven elements: Price, quality, attractiveness, brand image, emotions, advertising, and customer trust as effective elements on the sale of cosmetic and health products in the Iranian market. In order to measure the market situation and customers' opinions on the proposed elements, the measurement indicators of seven modern marketing mix elements that have been effective on sales are determined from the experts' point of view, and the validity of their measurement indicators is also confirmed with the content validity method and face validity from the experts' point of view. After determining the modern marketing mix elements from the experts' point of view, the effects of these elements in the actual sales market from the customers' point of view are confirmed by calculating their factor loading in structural equations through LISREL software, and their Cronbach's alpha coefficient and composite reliability in customer feedback are calculated and confirmed using SPSS software. Managers and owners of pharmacies can use them to measure the status of the elements and the level of satisfaction of their customers with the provided services. These findings are largely consistent with both domestic and international research. For instance, several studies conducted in the Iranian cosmetics market have reported that product quality, promotional strategies, and brand trust significantly influence customers’ purchase decisions in the Iranian cosmetics market. Likewise, research in the global beauty industry has emphasized that quality, price, and promotion are core determinants of consumer purchasing behavior in the global beauty industry. However, the present study also identified “people” and “physical evidence” as strong determinants of sales, which supports the transition from the traditional 4Ps model toward the extended marketing-mix frameworks (7Ps - 12Ps) discussed by Booms (15) and Goi (16). The slightly lower importance of “price” and “place” compared with other studies may be attributed to contextual factors in the Iranian pharmacy market, such as government-regulated pricing systems, limited product availability, and the advisory role of pharmacists in influencing purchase decisions. These comparisons demonstrate that while global marketing principles apply to the Iranian market, certain structural and cultural characteristics shape unique consumer behavior patterns.

ijpr-25-1-166280-s001.pdf

## Data Availability

The dataset presented in the study is available on request from the corresponding author during submission or after publication.

## Clinical Trial Registration Code:

This study did not involve any interventional research on humans or animals. The research was limited to voluntary responses from the statistical population (experts and customers of cosmetic products in Iranian pharmacies) and no clinical or laboratory procedures were performed. However, since this article is extracted from a PhD thesis in Pharmacy, it was reviewed and approved by the Ethics Committee of the Faculty of Pharmacy, Islamic Azad University, Tehran (approval code: IR.IAU.PS.REC.1403.371). Participation was completely voluntary, written informed consent was obtained from all respondents, and confidentiality was strictly observed.

## References

[1] Leonidou CN, Katsikeas CS, Morgan NA (2012). “Greening” the marketing mix: do firms do it and does it pay off?. J Acad Market Sci..

[2] Harini S, Yuningsih E, Gemina D (2020). Enhancing competitiveness of creative industry strategy with resource-based marketing mix.. 2nd International Seminar on Business, Economics, Social Science and Technology (ISBEST 2019)..

[3] Sang-Hyeon Y, Myung Soo K, Sang-Yeon S (2020). The Growth and Change of Korean Cosmetics Market in Distribution Structure.. J Distribution Sci..

[4] Brkanlić S, Sánchez-García J, Esteve EB, Brkić I, Ćirić M, Tatarski J (2020). Marketing Mix Instruments as Factors of Improvement of Students’ Satisfaction in Higher Education Institutions in Republic of Serbia and Spain.. Sustainability..

[5] Iyengar V, Chandrashekar SR (2023). Factors that affect consumer decision making on choosing sustainable cosmetic products: an empirical study.. Int J Progressive Res Engin Manag Sci..

[6] Andrean D, Anwar M (2021). The Effect of Perceived Quality, Brand Image, and Price Perception on Purchase Decision.. Proceedings of the 4th International Conference on Sustainable Innovation 2020-Accounting and Management (ICoSIAMS 2020)..

[7] Pourjabbar Z, Niazmand Badabi M, Salami F, Sadeghi N, Amini M, Hajimahmoodi M (2025). Quantification of Active Ingredients in Skin Lightening Creams Using HPLC Method in Iranian Market.. Jundishapur J Natural Pharm Prod..

[8] Ndofirepi E, Farinloye T, Mogaji E, Mogaji EM, Maringe F, Ebo Hinson R (2020). Marketing mix in a heterogenous higher education market.. Understanding the Higher Education Market in Africa..

[9] Lamasi WI, Santoso S (2022). The influence of promotion, product quality and brand image towards customer purchase decisions of Wardah cosmetic products.. Int J Res Business Soc Sci..

[10] Suryani M, Ms M (2022). The Influence of Brand Image, Price and Packaging Design on thePurchase Decision of Pixy Brand Cosmetics (Study on Users in Bandar Lampung).. Int J Regional Innovat..

[11] Katt F, Meixner O (2020). Is It All about the Price? An Analysis of the Purchase Intention for Organic Food in a Discount Setting by Means of Structural Equation Modeling.. Foods..

[12] Marín-Orantes TJ, del Río-Rama MDLC, Aguilasocho-Montoya D, Álvarez-García J (2025). Impact of marketing mix dimensions on the competitiveness of university centers.. Soc Sci Humanities Open..

[13] Jave-Chire M, Alvarez-Risco A, Guevara-Zavaleta V (2025). Footwear industry's journey through green marketing mix, brand value and sustainability.. Sustainable Futures..

[14] Salman D, Tawfik Y, Samy M, Artal-Tur A (2017). A new marketing mix model to rescue the hospitality industry: Evidence from Egypt after the Arab Spring.. Future Business J..

[15] Booms B, Donnelly JH, George WR (1981). Marketing strategies and organizational structures for service firms.. Marketing of services..

[16] Goi CL (2009). A Review of Marketing Mix: 4Ps or More?. Int J Market Stud..

[17] Chinsuvapala P (2017). Marketing Management.(15th global edition) Edinburgh: Pearson Education.. Kasem Bundit Journal..

[18] Adjie EA, Calista N, Muhtadiin RR, Handayani PW, Larasati PD (2023). User switching intention from E-marketplace to E-pharmacy: The Influence of push, pull, and mooring factors.. Inform Med Unlocked..

[19] Chakraborty D, Paul J (2023). Healthcare apps’ purchase intention: A consumption values perspective.. Technovation..

[20] Nugroho SDP, Rahayu M, Hapsari RDV (2022). The impacts of social media influencer’s credibility attributes on gen Z purchase intention with brand image as mediation.. Int J Res Business Soc Sci..

[21] Abdullah MF, Putit L, Teo CBC (2014). Impact of Relationship Marketing Tactics (RMT's) & Relationship Quality on Customer Loyalty: A Study within the Malaysian Mobile Telecommunication Industry.. Procedia - Soc Behav Sci..

[22] Khan Z (2021). Impact of Sales Promotion, Advertising and Direct Marketing on Sale of Cosmetic Products.. J Market Strateg..

[23] Andita DY, Najib MF, Zulfikar R, Purnamasari D (2021). The effect of celebrity endorser on purchase intention cosmetic product in millennial generation consumers.. J Market Innovat..

