# Supplementary material for: Determining Modern Marketing Mix Elements and Their Measurement Indicators for Selling Cosmetic and Health Products in Iranian Pharmacies: A Descriptive-Analytical Cross-sectional Study
Source: Iran J Pharm Res. 2026 Feb 12;25(1):e166280. doi: 10.5812/ijpr-166280 (PMC13558863; doi:10.5812/ijpr-166280)
Supplement: Supplementary file 1 [file ijpr-25-1-166280-s001.pdf]

Appendices

Appendix 1: Frequency of Likert scale responses to the questionnaire in the fuzzy Delphi method - first step – threshold

R	Questionnaire spectrum					Number of experts	Fuzzy value of each question			Defuzzified Value	Consensus percentage
	Very Low (1)	Low (2)	Average (3)	High (4)	Very High (5)		L	M	U		
1			0	2	8	10	3	4.76636	5	4.383178487	85
2			3	6	1	10	3	3.87405	5	3.937022591	80
3	1	8	1			10	1	2.57627	5	2.788133138	40
4		1	1		8	10	2	4.4238	5	3.961901055	80
5			1	2	7	10	3	4.54363	5	4.271815881	70
6	1	6	1	1	1	10	1	2.22505	5	2.612527332	60
7	6		1	3		10	1	1.98713	5	2.493566929	35
8			2	8		10	3	3.88657	4	3.693283316	90
9	1	8	1			10	1	2.57627	5	2.788133138	40
10			1		9	10	3	4.400.56	5	4.200279342	75
11			1	7	2	10	1	2.36311	5	2.681556862	35

12		1	2	7		10	2	3.75418	4	3.377087952	85

In Appendix 1, rows 3, 6, 7, 9, and 11 are eliminated as they have a consensus percentage of less than 70 percent. Other elements in the second step have a consensus of more than 70 percent and are selected as modern marketing mix elements that are effective in selling cosmetic and health products.W. Kendall coefficient (coefficient of agreement) is used to confirm this consensus. The null hypothesis measures the consensus between experts. In the second step test, the degree of freedom and the value of the chi-square are equal to 11.63 and the Kendall coefficient is 0.871 and the significance level is 0.137. Since it is not significant at an error level of less than 1% and the Kendall coefficient is higher than 0.5 and aims to 1, it can be stated with 99% confidence that the difference in the average ratings is not significant and there is consensus among the panel members.

Appendix 2: Test-retest reliability from the experts' perspective

R	Interview title	Total number of codes	Agreements	Disagreements	Test-retest reliability
1	First	79	25	11	63%
2	Second	41	14	8	68%
3	Total	120	39	19	65%

As shown in Appendix 2, the total number of codes in the 30-day period is 120, the number of agreements is 39, and the number of disagreements is 19. The test-retest reliability of the interviews conducted in the present study is 65 percent, and since this value is above 60 percent, the reliability of the items is confirmed.

Appendix 3: Analysis of variance by gender with all variables

		Total	Degree of freedom	Average	F	Level of Significance
Brand image	Between groups	.۱۹۷	۱	.۱۹۷	.۷۴۷	0.391
	Within groups	۱۴.۵۳۵	۳۲۷	.۲۶۴		
	Total	۱۴.۷۳۲	۳۲۸			
Attractiveness	Between groups	.۰۶۳	۱	.۰۶۳	.۱۵۳	0.697
	Within groups	۲۲.۵۰۷	۳۲۷	.۴۰۹		
	Total	۲۲.۵۷۰	۳۲۸			
Emotions	Between groups	.۹۲۵	۱	.۹۲۵	۲.۷۳۰	0.104
	Within groups	۱۸.۶۳۰	۳۲۷	.۳۳۹		
	Total	۱۹.۵۵۵	۳۲۸			
Quality / product	Between groups	.۱۰۷	۱	.۱۰۷	.۵۵۲	0.461
	Within groups	۱۰.۶۶۸	۳۲۷	.۱۹۴		
	Total	۱۰.۷۷۵	۳۲۸			
Trust	Between groups	.۵۳۸	۱	.۵۳۸	۲.۵۴۹	0.116
	Within groups	۱۱.۶۰۱	۳۲۷	.۲۱۱		
	Total	۱۲.۱۳۹	۳۲۸			
Advertising	Between groups	.۹۴۳	۱	.۹۴۳	۱.۶۳۱	0.207
	Within groups	۳۱.۷۸۷	۳۲۷	.۵۷۸		
	Total	۳۲.۷۳۰	۳۲۸			
Price	Between groups	.۰۰۰	۱	.۰۰۰	.۰۰۱	0.979

	Within groups	۱۰.۷۷۹	۳۲۷	.۱۹۶		
	Total	۱۰.۷۷۹	۳۲۸			

Is there a significant difference between the opinions of the samples in terms of gender?

H0: There is no significant difference.

H1: There is a significant difference.

The results of data analysis are presented using analysis of variance from SPSS software. As can be seen, the significance level is more than 5%, so there is no difference between the opinions of women and men in all factors.

Similar to the statistical analysis above that was conducted to test the difference in gender, a statistical test was also conducted for age, education level, and number of purchases, and the result, based on the significance level, was that these variables did not have a significant effect on the participants' opinions.

Appendix 4: Questionnaire Items and Sources

Marketing Mix Element	Example Items	Source / Origin
**Product**	Quality and safety of cosmetic products affect purchase decisions.	Kotler & Keller (2016)
	Product packaging and dermatological approval influence customer trust.	Researcher-designed
**Price**	Discounts and promotions increase willingness to buy.	Goi (2009)
	Perceived fairness of price affects customer satisfaction.	Researcher-designed
**Place (Distribution)**	Availability of products in pharmacies increases purchase frequency.	Lovelock & Wirtz (2016)

	Access to multiple pharmacy branches improves convenience.	Researcher-designed
**Promotion**	Staff recommendations affect customers' purchasing.	Researcher-designed
	Social media advertisements increase brand awareness.	Chaffey & Ellis-Chadwick (2020)
**People**	Pharmacist's knowledge and communication skills influence loyalty.	Kotler (2017)
	Professional appearance of staff builds credibility.	Researcher-designed
**Process**	Simplicity and speed of the purchasing process affect satisfaction.	Lovelock & Wirtz (2016)
	Offering free product samples improves trust.	Researcher-designed
**Physical Evidence**	Store layout and cleanliness influence perceived quality.	Kotler & Keller (2016)
	Lighting and product arrangement affect buying mood.	Researcher-designed

References for appendix (Appendix 4) :

1. Kotler, P., & Keller, K. L. (2016). Marketing Management (15th ed.). Pearson Education.
   → (Source for "Product" and "Price" indicators.)
2. Goi, C. L. (2009). A review of marketing mix: 4Ps or more? International Journal of Marketing Studies, 1(1), 2–15.
   → (Source for "Promotion" and general marketing mix framework.)
3. Lovelock, C., & Wirtz, J. (2016). Services Marketing: People, Technology, Strategy (8th ed.). Pearson Education.
   → (Source for "Process" and "People" dimensions.)
4. Zeithaml, V. A., Bitner, M. J., & Gremler, D. D. (2018). Services Marketing: Integrating Customer Focus Across the Firm (7th ed.). McGraw-

Hill Education.
→ (Source for “Physical Evidence” and “Service Quality.”)

5. Booms, B. H., & Bitner, M. J. (1981). Marketing strategies and organization structures for service firms. In J. H. Donnelly & W. R. George (Eds.), Marketing of Services (pp. 47–51). American Marketing Association.
→ (Original model introducing 7Ps — foundational reference.)
6. Chaffey, D., & Ellis-Chadwick, F. (2020). Digital Marketing: Strategy, Implementation and Practice (8th ed.). Pearson Education.
→ (Used to support updated marketing indicators in modern contexts.)
7. Hair, J. F., Black, W. C., Babin, B. J., & Anderson, R. E. (2019). Multivariate Data Analysis (8th ed.). Cengage Learning.
→ (Used for validation and factor analysis references.)
8. Parasuraman, A., Zeithaml, V. A., & Berry, L. L. (1988). SERVQUAL: A multiple-item scale for measuring consumer perceptions of service quality. Journal of Retailing, 64(1), 12–40.
→ (Source for customer satisfaction and service quality measures.)

Table 5: Determining expertise for conducting an interview

Years of experience	1 to 5 Years	6 to 10 Years	11 to 15 Years	16 to 20 Years	More than 20
Likert scale	Very low 1	Low 2	Average 3	High 4	Very high 5
Knowledge of cosmetic products					
Knowledge and experience in selling cosmetic products					
Score in both conditions	2	4	6	8	10
